# Variations within 3′-UTR of *MDM4* gene contribute to clinical outcomes of advanced non-small cell lung cancer patients following platinum-based chemotherapy

**DOI:** 10.18632/oncotarget.10771

**Published:** 2016-07-22

**Authors:** Yang Yang, Wen Gao, Xi Ding, Wen Xu, Di Liu, Bo Su, Yifeng Sun

**Affiliations:** ^1^ Department of Thoracic Surgery, Shanghai Chest Hospital, Shanghai Jiao Tong University, Shanghai, 200030, P.R. China; ^2^ Central Laboratory, Shanghai Pulmonary Hospital, Tongji University School of Medicine, Shanghai, 200433, P.R. China

**Keywords:** chemotherapy, non-small cell lung cancer, *MDM4*, platinum, single nucleotide polymorphisms

## Abstract

Single-nucleotide polymorphism (SNPs) in microRNA (miRNA)-binding sites may modulate the posttranscriptional regulation of gene expression and explain individual sensitivity to platinum agents. This study aimed to investigate the impact of SNPs located at 3′-untranslated region (UTR) of *MDM4* gene, on clinical outcomes of advanced non-small cell lung cancer (NSCLC) patients. Four SNPs were genotyped by using DNA from blood samples of advanced NSCLC patients (642 in the Discovery set and 330 in the Replication set) and were analyzed the relationships with clinical outcomes. Carriers with rs10900598 CC genotype and rs4245739 AC genotype showed increased overall survival (OS) than those with AA genotype (*P* = 0.017 and *P* = 0.037, respectively) in the Discovery set and after pooling results from the Replication set. A combined effect on survival of variant alleles was also concluded and validated. Stratification analysis revealed that the effect of *MDM4* SNPs was more pronounced in lung adenocarcinoma (LAC) subgroups. A reduced expression of the reporter gene for the C allele of rs4245739 was observed in NSCLC cells using luciferase reporter gene assays. Taken together, our results demonstrate that genetic variations in 3′-UTR of *MDM4* gene may influence outcomes of advanced NSCLC by miRNAs-mediated regulation.

## INTRODUCTION

In contrast to the steady increase in survival for most cancers, advances have been slow for non-small cell lung cancer (NSCLC), for which the 5-year survival rate is currently 18%. The low rate is partly contributed to that more than one-half of cases are diagnosed at a distant stage, for which 5-year relative survival is 4% [1]. Platinum agents combined with another kind of cytotoxic drugs have been the mainstay of treatment for advanced NSCLC regardless of various therapy responses between patients [2]. Therefore, novel molecular biomarkers are required to be identified for further development of individualized therapy.

*P53* signaling pathway has been demonstrated to be crucial in regulating chemotherapeutic effects of cancer treatment by initiating cellular responses such as DNA repair, cell cycle arrest, apoptosis, and senescence [3, 4]. Murine double minute 4 (*MDM4*), structurally homologous protein of murine double minute 2 (*MDM2*), contributes to cancer susceptibility and progression through its capacity to regulate transcriptional activity of the tumor suppressor *p53* [5]. The interaction between *MDM4* and *p53* in the regulation of cancer was investigated in several previous studies [6, 7]. As a key protein involved in the process of DNA damage response, inhibition of *p53* by *MDM2* and *MDM4* can result in delayed DNA repair [8–10]. However, later studies revealed a novel role of *MDM4* in *p53*-mediated intrinsic apoptotic pathway and the non-overlapping role in regulating *p53* activity with *MDM2* [11, 12]. Considering the previous conflicting reports about the function of *MDM4* in inhibiting *p53* activity, the relationship between *MDM4* and cancers needs to be further investigated.

In the last decades, an increasing number of researches had been focused on posttranscriptional regulation of gene expression by microRNAs (miRNAs) and their potential effects on the development and prognosis of cancer. miRNAs are small, single-stranded, non-coding RNAs that regulate gene expression through canonical base pairing with complementary seed sequence located in the 3′-untranslated region (UTR) of target mRNAs [13]. Association between genetic variations in this region of *MDM4* gene and cancers had been investigated in previous studies. The A-allele of *MDM4* rs4245739A>C (SNP34091) was associated with increased expression of *MDM4* mRNA and protein levels by miR-191-5p regulation in ovarian carcinomas and sensitivity for chemotherapy [14]. Another study reported the regulation of *MDM4* rs4245739 polymorphism by two miRNAs (miR-191-5p and miR-887-3p), and the A-allele of rs4245739 may be associated with an increased risk for prostate cancer [15].

However, in spite of the well-known role in tumor pathogenesis and progression, few genetic variants of *MDM4* had been found to be associated with NSCLC. To fill this gap, we chose to focus on potential miRNAs-binding *MDM4* polymorphisms in order to detecting variants that alter treatment efficacy of platinum-based chemotherapy. In the present study, four SNPs located at the 3′-UTRs of *MDM4* gene were selected for further investigation.

## RESULTS

### Patient characteristics and genotype frequencies

Demographic and clinical characteristics of patients in two study sets were presented in Table 1. In the Discovery set, 456 (71.0%) were male and 382 (59.5%) were smokers. All patients were diagnosed with advanced inoperable NSCLC and had received platinum-based chemotherapy, with 40.8% of stage III and 59.2% of stage IV. Variables of the Replication set showed no statistically differences when compared with the Discovery set. Genotype frequencies of four *MDM4* polymorphisms were in Hardy–Weinberg equilibrium (*P* > 0.05). The investigated SNPs were classified by models including genotypic, dominant, recessive, or additive. There was no significant difference in genotype distributions of all selected SNPs according to clinical factors (Supplementary Table S1).

**Table 1:** Basic characteristics of patients from the two cohorts

Variables	Discovery set (N, %)(n=642)	Replication set (N, %)(n=330)	*χ*^2^	*P*^a^
Age (years)				
< 58	333 (51.9)	171 (51.8)	0.001	0.988
≥ 58	309 (48.1)	159 (48.2)		
Gender				
Male	456 (71.0)	234 (70.9)	0.001	0.969
Female	186 (29.0)	96 (29.1)		
Smoking history				
Non-smokers	260 (40.5)	144 (43.6)	0.884	0.347
Smokers	382 (59.5)	186 (56.4)		
ECOG PS				
0-1	593 (92.4)	297 (90.0)	1.582	0.209
2	49 (7.6)	33 (10.0)		
Chemotherapy				
NP/NC	236 (36.8)	115 (34.8)	1.126	0.771
GP/GC	174 (27.1)	95 (28.8)		
TP/TC	192 (29.9)	95 (28.8)		
DP/DC	40 (6.2)	25 (7.6)		
TNM stage				
III	262 (40.8)	119 (36.1)	2.063	0.151
IV	380 (59.2)	211 (63.9)		
Histological type				
Adeno	398 (62.0)	213 (64.5)	2.057	0.358
SQC	147 (22.9)	66 (20.0)		
Others	97 (15.1)	51 (15.5)		

Adeno adenocarcinoma, SQC squamous cell carcinoma, ^a^ *p*-values derived from *χ*^2^ test.

### Survival analysis

#### Discovery set

The median overall survival (OS) and progression-free survival (PFS) (53 patients were excluded for PFS analysis because of incomplete information) for all the patients was 19.27 (95% CI, 17.64-20.90) and 10.07 (95% CI, 8.61-11.53) months, respectively. Patients with TNM stage IV and infrequently histological subtypes had significant worse OS (aHR=1.28, 95% CI, 1.06-1.54, *P* = 0.012; and aHR=1.35, 95% CI, 1.03-1.76, *P* = 0.027; respectively) compared to patients with TNM stage III and adenocarcinoma. In addition, ECOG PS was related to PFS and patients with higher scores showed a higher risk of recurrence or metastasis (aHR=1.70, 95% CI, 1.16-2.49, *P* = 0.006), whereas other clinical factors were not independent predictive factors (Supplementary Table S2).

**Figure 1:**
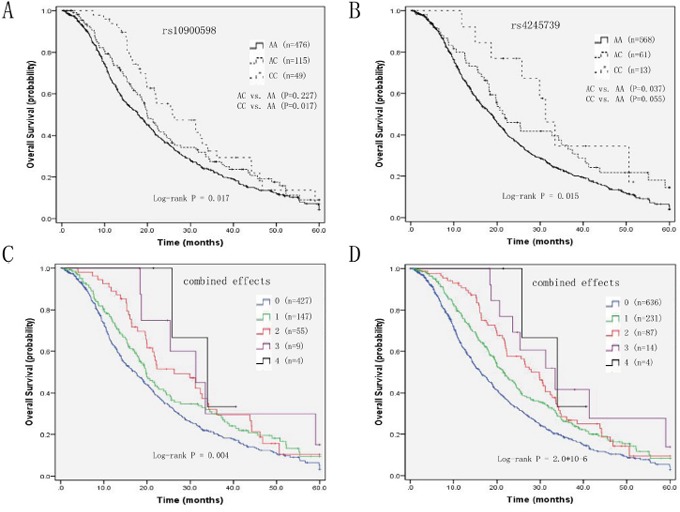
Kaplan-Meier curves of **A**. OS and rs10900598 in Discovery set, **B**. OS and rs4245739 in Discovery set, **C**. number of variant alleles from two SNPs in Discovery set, and **D**. number of variant alleles from two SNPs in Pooled populations.

Using *MDM4* genotypes as categorical variables, two SNPs (rs10900598 and rs4245739) were significantly associated with OS (*P* = 0.017 and *P* = 0.015), but not with PFS (*P* = 0.429 and *P* = 0.302; Supplementary Table S3). For rs10900598, HRs were significantly lower for individuals with CC genotype compared with AA genotype (aHR=0.65, 95% CI, 0.46-0.93, *P* = 0.017), whereas AC genotype showed no significance after adjusting for clinical factors. In the dominant and recessive model, the association between C variant allele and better survival was concluded (for CC+AC genotype HR, 0.79; 95% CI, 0.64-0.97; *P* = 0.026; for CC genotype HR, 0.67; 95% CI, 0.47-0.95; *P* = 0.025). In contrast, carriers of rs4245739 AC genotype were associated with prolonged survival (aHR=0.71, 95% CI, 0.52-0.98, *P* = 0.037), whereas patients with CC genotype didn't remain significant after clinical factors were adjusted. Although not reaching statistical significance, rs4245739 CC genotype showed a similar trend of association with OS. In addition, in the dominant model, C allele carriers had better survival compared with A allele carriers (for C allele HR, 0.67; 95% CI, 0.50-0.90; *P* = 0.007) (Table 2). Furthermore, we included age, TNM stage, histological type, rs10900598 dominant model, and rs4245739 dominant model in the multivariate Cox's regression analysis. The results suggested that TNM stage (*P*= 0.008), histological type (*P*= 0.014), rs10900598 dominant model (*P*= 0.023), and rs4245739 dominant model (*P*= 0.007) were independent predictive factors for OS (Table 3). The Kaplan-Meier curves of OS according to *MDM4* SNPs were shown in Figure 1 (Figure 1A for rs10900598; Figure 1B for rs4245739).

**Table 2:** Association between *MDM4* SNPs and OS of patients in each set and in pooled populations

SNPs	Discovery set					Replication set					Pooled				
	N	mOS (95% CI) (m)^a^	*P*_L-R_	aHR (95% CI)^b^	*P*^b^	N	mOS (95% CI) (m)^a^	*P*_L-R_	aHR (95% CI)^b^	*P*^b^	N	mOS (95% CI) (m)^a^	*P*_L-R_	aHR (95% CI)^b^	*P*^b^
**rs10900598**															
AA	476	17.80 (15.44-20.16)	0.017	Ref.		233	16.40 (13.64-19.16)	0.007	Ref.		709	17.57 (15.93-19.21)	2.15* 10^-4^	Ref.	
AC	115	20.00 (17.45-22.55)		0.86 (0.68-1.10)	0.227	73	24.03 (17.85-30.22)		0.77 (0.57-1.06)	0.107	188	21.27 (18.11-24.42)		0.83 (0.69-1.00)	0.055
CC	49	25.77 (15.92-35.61)		0.65 (0.46-0.93)	0.017	24	29.77 (23.57-35.96)		0.49 (0.29-0.82)	0.007	73	27.43 (22.35-32.51)		0.60 (0.45-0.80)	0.001
dominant															
CC+AC	164	21.90 (18.73-25.07)	0.008	0.79 (0.64-0.97)	0.026	97	25.60 (21.17-30.03)	0.004	0.68 (0.51-0.91)	0.008	261	22.63 (20.07-25.19)	1.59* 10^-4^	0.75 (0.64-0.89)	0.001
AA	476	17.80 (15.44-20.16)		Ref.		233	16.40 (13.64-19.16)		Ref.		709	17.57 (15.93-19.21)		Ref.	
recessive															
CC	49	25.77 (15.92-35.61)	0.023	0.67 (0.47-0.95)	0.025	24	29.77 (23.57-35.96)	0.012	0.52 (0.31-0.88)	0.013	73	27.43 (22.35-32.51)	0.001	0.62 (0.47-0.83)	0.001
AA+AC	591	18.67 (16.97-20.36)		Ref.		306	17.90 (15.67-20.13)		Ref.		897	18.30 (16.91-19.69)		Ref.	
additive		NA	NA	0.82 (0.71-0.96)	0.011		NA	NA	0.73 (0.59-0.90)	0.003		NA	NA	0.79 (0.70-0.90)	1.67* 10^-4^
**rs4245739**															
AA	568	18.67 (16.83-20.50)	0.015	Ref.		294	17.70 (15.50-19.91)	0.018	Ref.		862	18.00 (16.60-19.40)	0.001	Ref.	
AC	61	21.27 (15.07-27.47)		0.71 (0.52-0.98)	0.037	30	27.90 (19.76-36.04)		0.71 (0.46-1.10)	0.121	91	24.47 (18.68-30.26)		0.71 (0.55-0.91)	0.008
CC	13	31.27 (26.49-36.05)		0.50 (0.25-1.02)	0.055	6	30.63 (16.29-44.97)		0.49 (0.18-1.35)	0.166	19	31.27 (27.17-35.36)		0.50 (0.28-0.89)	0.019
dominant															
CC+AC	74	25.37 (15.95-34.78)	0.005	0.67 (0.50-0.90)	0.007	36	29.80 (23.58-36.02)	0.006	0.67 (0.44-1.01)	0.051	110	26.40 (20.56-32.24)	2.06* 10^-4^	0.67 (0.53-0.85)	0.001
AA	568	18.67 (16.83-20.50)		Ref.		294	17.70 (15.50-19.91)		Ref.		862	18.00 (16.60-19.40)		Ref.	
recessive															
CC	13	31.27 (26.49-36.05)	0.066	0.52 (0.26-1.05)	0.069	6	30.63 (16.29-44.97)	0.085	0.52 (0.19-1.41)	0.198	19	31.27 (27.17-35.36)	0.017	0.52 (0.29-0.93)	0.026
AA+AC	629	19.10 (17.46-20.74)		Ref.		324	19.03 (16.44-21.70)		Ref.		953	19.07 (17.67-20.46)		Ref.	
additive		NA	NA	0.71 (0.56-0.90)	0.005		NA	NA	0.71 (0.50-0.99)	0.043		NA	NA	0.71 (0.59-0.86)	4.99* 10^-4^

OS overall survival, m months, Ref. reference, NA not available, HR hazard ratio, CI confidence interval, *P*_L-R_ Log-Rank *P*;

^a^ survival derived from Kaplan–Meier analysis;

^b^ HRs, 95% CIs and their corresponding *p*-values were calculated using multivariate Cox proportional hazard models, adjusted for all clinical factors.

**Table 3:** Multivariate Cox's regression analysis of prognostic factors for overall survival in Discovery set

Variables	HR (95% CI)	*P*
Age (≥ 58 vs. < 58)	1.21 (1.01-1.45)	0.053
TNM stage (IV vs. III)	1.29 (1.07-1.55)	0.008
Histological type		0.014
Adeno	Ref.	
SQC	1.26 (1.00-1.58)	0.049
Others	1.40 (1.08-1.82)	0.011
rs10900598		
dominant	0.78 (0.64-0.97)	0.023
rs4245739		
dominant	0.67 (0.50-0.90)	0.007

Adeno adenocarcinoma, SQC squamous cell carcinoma. All the variables yielding *P*-values< 0.1 in the univariate analysis were used for multivariate Cox's regression.

To better define the influence of *MDM4* SNPs on OS, we performed a subgroup analysis stratified by clinical factors. The results suggested that the above associations were more pronounced in lung adenocarcinoma (LAC) subjects. Patients with rs10900598 CC genotype and rs4245739 AC genotype had marginally prolonged OS than those with wild-type AA genotype (31.20 vs. 19.10, *P* = 0.039; and 32.70 vs. 19.90, *P* = 0.042; respectively). Subgroup multivariate Cox's regression analysis revealed that relevant aHRs were 0.64 and 0.67, respectively (*P* = 0.048 and *P* = 0.047). In addition, the above significant models of rs10900598 and rs4245739 also showed similar relationship with OS in LAC patients (for dominant, additive model of rs10900598 aHR=0.73, 95% CI, 0.55-0.95, *P* = 0.020, and aHR=0.79, 95% CI, 0.65-0.96, *P* = 0.017, respectively; for dominant, additive model of rs4245739 aHR=0.65, 95% CI, 0.45-0.93, *P* = 0.020, and aHR=0.69, 95% CI, 0.50-0.94, *P* = 0.019, respectively) (Supplementary Table S4).

### Replication set and pooled analysis

The median OS and PFS (4 patients were excluded for PFS analysis because of incomplete information) for the Replication set was 19.07 (95% CI, 16.44-21.70) and 7.33 (95% CI, 5.95-8.71) months, respectively. In this second group, we concluded that rs10900598 CC genotype was associated with better OS (aHR=0.49, 95% CI, 0.29-0.82, *P* = 0.007). The dominant model (*P* = 0.008), recessive model (*P* = 0.013), and additive model (*P* = 0.003) of rs10900598 also showed statistically significant association. For rs4245739, the Kaplan-Meier plots of OS were significant in genotypic and dominant model (*P* = 0.018 and *P* = 0.006). However, we failed to detect the differences of OS in patients with rs4245739 polymorphisms except for the additive model after adjusting for all other covariates (aHR=0.71, 95% CI, 0.50-0.99, *P* = 0.043) (Table 2).

The pooled analysis of two sets confirmed the previously observed association with OS for rs10900598 (for CC+AC genotype HR, 0.75, 95% CI, 0.64-0.89, *P* = 0.001; for CC genotype HR, 0.62; 95% CI, 0.47-0.83; *P* = 0.001; for additive model HR, 0.79, 95% CI, 0.70-0.90; *P* = 1.67*10^-4^), as well as for rs4245739 (for CC+AC genotype HR, 0.67, 95% CI, 0.53-0.85, *P* = 0.001; for CC genotype HR, 0.52; 95% CI, 0.29-0.93; *P* = 0.026; for additive model HR, 0.71, 95% CI, 0.59-0.86; *P* = 4.99*10^-4^) (Table 2).

### Combined effects of the rs10900598 and rs4245739 on survival

We performed an exploratory analysis to investigate the combined effects of *MDM4* rs10900598 and rs4245739 polymorphisms, which had a significant association with OS in the individual SNP analysis, on survival outcomes. When we grouped patients according to the number of variant alleles (i.e., 0, 1, 2, 3, or 4), OS was increased with increasing number of variant alleles in a stepwise manner. In the Discovery set, OS for patients with four variant alleles was 33.93 (95% CI, 20.86-47.00) as compared with 31.27 (95% CI, 16.75-45.78), 26.30 (95% CI, 16.69-35.91), 19.77 (95% CI, 16.97-22.56), and 17.20 (95% CI, 14.85-19.55) months for those with three, two, one, and zero variant alleles, respectively (*P* = 0.004, Figure 1C). Multivariate Cox's proportional hazard analysis showed that, compared with the reference group of patients with zero variant allele, those with one, two, three and four variant alleles had aHRs of 0.80, 0.67, 0.44, and 0.36 respectively (*P*_trend_ =0.010; Table 5). In the pooled analysis, the Kaplan-Meier plots and log-rank tests confirmed the combined effects of the two SNPs on survival (*P* = 2.0*10^-6^, Figure 1D). In addition, compared with patients with zero variant allele, those with one, two, three and four variant alleles had aHRs of 0.76, 0.62, 0.42, and 0.35 respectively (*P*_trend_ = 3.3*10^-5^) (Table 4).

**Table 5:** Correlations of significant *MDM4* SNPs with chemotherapy efficacy in Discovery set

Variables	ORR (CR + PR)					DCR (CR + PR + SD)				
	N (%)	N (%)*χ*^2^	N (%)*P*^a^	N (%)OR (95% CI)^b^	N (%)*P*^b^	N (%)N (%)	N (%)*χ*^2^	N (%)*P*^a^	N (%)OR (95% CI)^b^	N (%)*P*^b^
**rs10900598**										
AA	79 (16.6)	7.844	0.020	Ref.		379 (79.6)	2.799	0.247	Ref.	
AC	23 (20.0)			0.80 (0.47-1.36)	0.412	99 (86.1)			0.65 (0.36-1.15)	0.139
CC	16 (32.7)			0.35 (0.18-0.69)	0.003	38 (77.6)			1.15 (0.56-2.35)	0.712
dominant										
CC+AC	39 (23.8)	4.186	0.041	0.62 (0.39-0.96)	0.034	137 (83.5)	1.197	0.274	0.78 (0.49-1.26)	0.317
AA	79 (16.6)			Ref.		379 (79.6)			Ref.	
recessive										
CC	16 (32.7)	7.131	0.008	0.37 (0.19-0.72)	0.003	38 (77.6)	0.321	0.571	1.24 (0.61-2.53)	0.561
AA+AC	102 (17.3)			Ref.		478 (80.9)			Ref.	
additive		NA	NA	0.64 (0.47-0.88)	0.005		NA	NA	0.93 (0.66-1.29)	0.646
**rs4245739**										
AA	99 (17.4)	4.140	0.126	Ref.		457 (80.5)	0.471	0.790	Ref.	
AC	16 (26.2)			0.53 (0.28-1.01)	0.050	51 (83.6)			0.79 (0.38-1.62)	0.516
CC	4 (30.8)			0.54 (0.15-1.88)	0.331	10 (76.9)			1.34 (0.35-5.12)	0.672
dominant										
CC+AC	20 (27.0)	3.994	0.046	0.53 (0.30-0.95)	0.033	61 (82.4)	0.164	0.686	0.87 (0.46-1.66)	0.674
AA	99 (17.4)			Ref.		457 (80.5)			Ref.	
recessive										
CC	4 (30.8)	1.315	0.251	0.58 (0.17-2.02)	0.388	10 (76.9)	0.121	0.728	1.37 (0.36-5.23)	0.647
AA+AC	115 (18.3)		Ref.			508 (80.8)			Ref.	
additive		NA	NA	0.63 (0.40-0.99)	0.046		NA	NA	0.95 (0.57-1.60)	0.857

ORR objective response rate, DCR disease control rate, CR complete response, PR partial response, SD stable disease, Ref. reference, NA not available, OR, odds ratio; CI, confidence interval;

^a^ *p*-value derived from *χ*^2^ test;

^b^ ORs, 95% CIs and their corresponding *p*-values were calculated using multivariate logistic regression analysis, adjusted for all clinical factors.

**Table 4:** Combined effects of *MDM4* rs10900598 and rs4245739 in Discovery set and in pooled populations

No. ofVariant Alleles	Discovery set					Pooled				
	N (%)	mOS (95% CI) (m)^a^	*P*_L-R_	aHR (95% CI)^b^	*P*^b^	N (%)	mOS (95% CI) (m)^a^	*P*_L-R_	aHR (95% CI)^b^	*P*^b^
0	427 (66.5)	17.20 (14.85-19.55)	0.004	Ref.		636 (65.4)	16.27 (14.45-18.08)	2.0*10^-6^	Ref.	
1	147 (22.9)	19.77 (16.97-22.56)		0.80 (0.64-1.00)	0.045	231 (23.8)	21.47 (18.69-24.24)		0.76 (0.63-0.90)	0.002
2	55 (8.6)	26.30 (16.69-35.91)		0.67 (0.48-0.93)	0.016	87 (9.0)	27.90 (23.42-32.38)		0.62 (0.48-0.80)	3.27* 10^-4^
3	9 (1.4)	31.27 (16.75-45.78)		0.44 (0.19-1.00)	0.029	14 (1.4)	33.47 (21.18-45.76)		0.42 (0.22-0.81)	0.016
4	4 (0.6)	33.93 (20.86-47.00)		0.36 (0.09-1.47)	0.015	4 (0.4)	33.93 (20.86-47.00)		0.35 (0.09-1.40)	0.010
				*P*_trend_ = 0.010					*P*_trend_ = 3.3*10^-5^	

OS overall survival, m months, Ref. reference, HR hazard ratio, CI confidence interval, *P*_L-R_ Log-Rank *P*;

^a^ survival derived from Kaplan–Meier analysis;

^b^ HRs, 95% CIs and their corresponding *p*-values were calculated using multivariate Cox proportional hazard models, adjusted for all clinical factors.

### *MDM4* polymorphisms and chemotherapy efficacy

Significant association with objective response rate (ORR) other than disease control rate (DCR) was manifested in 642 NSCLC patients of Discovery set (Table 5). Association with ORR for rs10900598 (for CC+AC genotype HR, 0.62, 95% CI, 0.39-0.96, *P* = 0.034; for CC genotype HR, 0.37; 95% CI, 0.19-0.72; *P* = 0.003; for additive model HR, 0.64, 95% CI, 0.47-0.88; *P* = 0.005), as well as for rs4245739 (for CC+AC genotype HR, 0.53, 95% CI, 0.30-0.95, *P* = 0.033; for additive model HR, 0.63, 95% CI, 0.40-0.99; *P* = 0.046). However, the relationship with ORR was not proved significant in terms of rs4245739 CC genotype (*P* = 0.388).

### Haplotype analysis

Pairwise linkage disequilibriums for the four SNPs are presented, respectively, in Supplementary Table S5. Since the LD among these four SNPs of *MDM4* was incomplete in all subjects of the Discovery set, we hypothesized that variant haplotypes may have an influence on survival. Hence, we inferred twelve *MDM4* haplotypes, three of which had a frequency more than 0.10 and were selected for further analysis. The association between *MDM4* haplotypes with OS in the Discovery set was summarized in Supplementary Table S6. However, no significant difference in OS between patients with one or two copies and zero copies of *MDM4* haplotypes (G-A-A-A, G-A-C-A, and A-A-C-C) was concluded.

### *In vitro* assay

**Figure 2:**
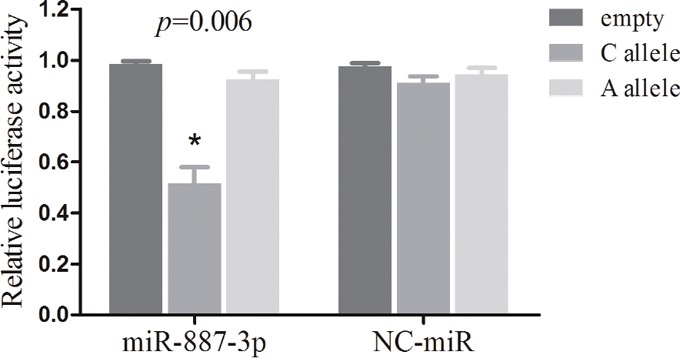
Dual luciferase reporter gene assays with constructs containing different alleles of *MDM4* 3′-UTR region or pGL3-control vector (empty) were co-transfected with miR-887-3p mimics or NC-miR in the NSCLC A549 cells The data are normalized to the luciferase activity observed with the empty vector, which is set to 1. All experiments were performed in triplicates and each value represents mean ± SD. * *P* < 0.05 compared with the empty control construct by *t*-tests. NC-miR, negative control miRNAs; NSCLC, non-small cell lung cancer.

We used Mirnsnpscore software (http://www.bigr.medisin.ntnu.no/mirsnpscore/), based on *in silico* predictions of SNP effects on miRNA-based gene regulation, to assess the regulatory effect of the two significant SNPs. Mirnsnpscore could sorts selected SNPs and calculates a DeltaS score that reflects the potential effect on miRNA regulation. In particular, rs4245739 had the highest DeltaS score among the selected SNPs in the 3′-UTR of *MDM4* gene (DeltaS = 0.60 for miR-887-3p), while rs10900598 only had a DeltaS score of 0.17 for miR-3173. Hence, we examined whether there is an allele-specific effect of rs4245739 polymorphism on *MDM4* expression in NSCLC cells by the regulation of miR-887-3p by *in vitro* assay. The results indicated that miR-887-3p can significant lower luciferase activities in A549 cells co-transfected with rs4245739 C-allelic reporter constructs compared to negative control in all the experiments performed. Combining results from all the experiments, the average luciferase expression was reduced to 54.1% when considering the control groups as reference (*P* = 0.006, Figure 2).

## DISCUSSION

The principal finding of this trial is that two SNPs (rs10900598 and rs4245739), located at 3′-UTR of *MDM4* gene, contribute to clinical outcome of advanced NSCLC patients treated with platinum-based chemotherapy. In addition, the inhibition of rs4245739 CC genotype on gene expression by miR-887-3p regulation is demonstrated through *in vitro* assay. Given the role of *MDM4* as a key inhibitor of *p53* [16], it is biologically plausible that the *MDM4* SNPs may correlate with chemotherapy efficacy of DNA-damaging anti-cancer drugs.

The crucial role of *p53* as “molecular police” of genome integrity detection had been confirmed and clarified long ago. Modern pharmacology researches showed that anticancer drugs such as platinum could induce DNA damage of cancer cells and result in the activation of cell cycle checkpoints of G1-S and G2-M phases, which is followed by cell cycle arrest [17]. Then, the process of DNA repair, including mismatch repair (MMR) and nucleotide excision repair (NER) is started. Abrogation of cell cycle arrest will force cells into mitotic catastrophe or apoptosis and cell death [18]. Since the dysregulation of DNA repair is involved in chemotherapy resistance to NSCLC, it has been speculated that mutations of potentially targeted oncogenes or tumor suppressor genes should also be taken into consideration [19]. Therefore, variations in the DNA repair capacity including *p53* dysregulation by *MDM4* abnormal expression may play a role on clinical outcomes of advanced NSCLC patients.

miRNAs have been shown to modulate diverse biological processes, including DNA repair, by interacting with 3′-UTRs of potential targeted genes [20]. In addition, Several previous studies including genome-wide association study (GAWS) had confirmed the relationship between *MDM4* 3′-UTR SNPs and susceptibility of breast cancer, esophageal squamous cell carcinoma, and small cell lung cancer in different ethnic populations [21–23]. These results elucidated underlying mechanism that variations of *MDM4* 3′-UTR SNPs could alter the binding with specific miRNAs and modulate gene expression, ultimately affect cancer development. Beyond that, however, 3′-UTR of gene also harbors several regulatory motifs that determine mRNA turnover, stability, and localization together with different RNA-interacting factors [24]. To our knowledge, our study is the relatively largest investigation of *MDM4* gene SNPs in NSCLC prognosis.

In this present study, we demonstrated that *MDM4* rs10900598 and rs4245739 SNPs (located at 3′-UTR) were associated with increased overall survival in advanced NSCLC patients, and the results were even more apparent in LAC patients. This finding may be explained that patients with squamous cell carcinoma, adenosquamous carcinoma, or other histological types of lung cancer sometimes have complex somatic mutations driving treatment resistance when compared with adenocarcinoma, which may make that modest effect of genotypes on *MDM4* expression is overwhelmed. In the opinion of my team, any significant association for rs4245739 CC genotype may be weak due to that genotype heterogeneity and relatively small sample size make it easy to be masked. When another replication set was added, the above results were validated and a more powerful statistical significance was reached in the pooled analysis. In addition, we found that variant genotypes of rs10900598 and rs4245739 SNPs showed preferable treatment response, which may contribute to better survival than patients with wild-type genotypes.

The present study has several strengths. We used two relatively large cohorts from four independent oncological departments for the two-stage association analysis between *MDM4* SNPs and clinical outcome of advanced NSCLC patients. In addition, we also evaluated multiple *MDM4* polymorphisms individually classified by models including genotypic, dominant, recessive, or additive and collectively as haplotypes. However, we acknowledge that there are several limitations of the present study. First is the retrospective nature of the study. Second, although it was rational to conduct a pooled analysis with two similar databases, we cannot ignore certain differences such as different time interval of follow-up and the collection of clinical information. Third, the functional relevance of rs10900598 polymorphism on miRNAs-mediated *MDM4* regulation was not assessed because of the low DeltaS score. Further *in vitro* functional assays are needed to verify this genetic variant of *MDM4* gene as predictive biomarkers and to find the underlying biologic mechanisms.

In conclusion, we have identified SNPs in the 3′-UTR of *MDM4* gene among which rs4245739 and rs10900598 were associated with clinical outcome of advanced NSCLC patients treated with platinum-based chemotherapy, whereby the minor allele carriers have better overall survival and treatment response. Further studies are needed to investigate underlying precise mechanisms by which the *MDM4* SNPs affect outcome of advanced NSCLC patients.

## MATERIALS AND METHODS

### Study population

The Discovery set included 642 patients with histologically confirmed late-stage (III–IV) NSCLC who had received first-line platinum-based chemotherapy between March 2005 and January 2010 from oncological departments of Shanghai Zhongshan Hospital, Shanghai Chest Hospital, and Shanghai Changhai Hospital. Positive hits from the Discovery set were validated in patients with advanced NSCLC from a Replication cohort. This dataset included 330 newly diagnosed and histologically confirmed NSCLC cases in Shanghai Pulmonary Hospital between June 2010 and May 2013. Blood samples from all subjects were collected at the time of diagnosis prior to chemotherapy treatment.

All subjects were informed and provided written consents to participate in the study and to approve the use of their biological samples for genetic analysis. The study was approved by the Medical Ethics Committee of each participating institution.

### Follow-up

Patients were interviewed face-to-face to collect demographic data including age, gender and smoking status. Those who had a low smoking frequency (<1 cigarette per day) and duration (<1 year) in their lifetime were defined as non-smokers. Clinical and follow-up information was obtained from medical records. Performance status was determined based on the Eastern Cooperative Oncology Group (ECOG) scale prior to treatment [25]. Patients’ response to chemotherapy was assessed after the first two or three cycles and determined by the Response Evaluation Criteria in Solid Tumors (RECIST) criteria 1.1 [26]. The disease control rate (DCR) included complete response (CR), partial response (PR) and stable disease (SD). The objective response rate (ORR) consisted of complete response (CR) and partial response (PR). Follow-up was performed per three months from the time of enrollment till death or the latest follow-up. OS was calculated as the first day to receive chemotherapy treatment to the day of death or to the latest follow-up. PFS was defined as the time from first treatment to the date of disease progression, death or last follow-up.

### Chemotherapy regimens

All the patients enrolled in the study were given first-line platinum-based chemotherapy. The detailed chemotherapeutic regimens have been described previously [27]. All chemotherapeutic drugs were given intravenously, and the patients were treated for 2 to 6 cycles.

### *MDM4* polymorphisms selection and genotyping

Genotype data for SNPs of *MDM4* gene were collected from two independent Chinese populations. We used the public HapMap SNP database (http://www.hapmap.org/) to identify the *MDM4* tagging SNPs within 3′-UTR on chromosome 1q32 by using the tagger algorithm with a minor allele frequency (MAF) cutoff of 0.05 and a correlation coefficient (r^2^) threshold of 0.8. Genomic DNA was extracted from peripheral blood lymphocytes using the Human Whole Blood Genomic DNA Extraction Kit (Qiagen, Valencia, CA). For each SNP, genotyping was performed using the TaqMan Pre-Designed SNP Genotyping Assays (Applied Biosystems, Foster City, CA) following manufacturer's instructions. Approximately 15% of the samples were randomly selected for repeat genotyping by a different investigator, and the results were entirely concordant. SNPs were excluded from further analysis if they had call rates less than 95% or Hardy–Weinberg equilibrium *p*-values or MAFs less than 0.05.

### Plasmid construction

The *MDM4* rs4245739A and rs4245739C allelic reporter constructs were generated by amplifying a 640-bp fragment of the *MDM4* 3′-UTR region from subjects homozygous for the rs4245739 AA or rs4245739 CC genotype. PCR primers used were as follows: 5′ -AACTCTAGAGGTAGTACGAACATAAAAATGC- 3′, and 5′- AACTCTAGACTGCATAAAGTAATCCATGG- 3′, which were also designed to include the *Xba* I restriction site sequence (T^CTAGA). The PCR products were digested with *Xba* I (NEB Inc.) and ligated, respectively, into an appropriately digested pGL3-control vector (Promega). The constructs were designated as pGL3-rs4245739A and pGL3-rs4245739C, respectively. The inserts were confirmed by DNA sequencing.

### Luciferase reporter assay

A firefly luciferase reporter plasmid (pGL3-control, pGL3-rs4245739A or pGL3-rs4245739C), a renilla luciferase vector (pRL-SV40, Promega) plus 100 nmol of synthesized miR-887 mimics or negative control (Genepharma, China) were co-transfected into A549 cells with Lipofectamine 2000 (Invitrogen, CA). The relative luciferase units (RLU) were determined at 48h after transfection using the dual-luciferase reporter assay system (Promega; Madison, WI, USA) according to the manufacturer's instructions. Three independent experiments were performed and each was done in triplicate. Fold change was reported by setting the scores of the pGL3-control groups as one and normalizing the scores in other groups.

### Statistical analysis

All statistical analyses were accomplished using SPSS version 20.0 (SPSS Inc., Chicago, IL, USA). Demographic and clinical characteristics were compared across genotype using Chi-square test for categorical variables. Hardy–Weinberg equilibrium was tested by comparing the observed genotype frequencies using a goodness-of-fit *χ* ^2^ test. For pairwise linkage disequilibrium analysis, D' and r^2^ for each pair of SNPs were calculated by the Haploview Software [28]. Individual haplotype frequency was estimated based on the Bayesian algorithm using the PHASE 2.0 program (version 2.0.2) [29]. Logistic regression was used for calculating odds ratio (OR), 95% confidence intervals (CI) and trend test. Survival curves were computed according to the Kaplan-Meier method. Cox's Proportional Hazard Model was used to test the relationship between SNPs and survival of patients. For all SNPs with significant *p* values per genotype, the dominant, recessive and additive models were also calculated. All statistical analyses were two-sided and the differences were considered as statistically significant at *p* < 0.05.

## SUPPLEMENTARY TABLES










