# Supplementary material for: Variations within 3′-UTR of MDM4 gene contribute to clinical outcomes of advanced non-small cell lung cancer patients following platinum-based chemotherapy
Source: Oncotarget. 2016 Jul 22;8(10):16313–24. doi: 10.18632/oncotarget.10771 (PMC5369965; doi:10.18632/oncotarget.10771)
Supplement: Supplementary file 1 [file oncotarget-08-16313-s001.pdf]

Variations within 3'-UTR of *MDM4* gene contribute to clinical outcomes of advanced non-small cell lung cancer patients following platinum-based chemotherapy

SUPPLEMENTARY TABLES

Supplementary Table S1: Distribution of *MDM4* genotypes according to clinical factors in Discovery set

See Supplementary File 1

Supplementary Table S2: Clinical characteristics associated with OS and PFS of patients in each set and in pooled populations

See Supplementary File 2

Supplementary Table S3: OS and PFS in relation to *MDM4* genotype distributions in Discovery set

See Supplementary File 3

Supplementary Table S4: Association between *MDM4* SNPs and OS in Discovery set stratified by tumor histology

See Supplementary File 4

Supplementary Table S5: Linkage disequilibrium (D’ and r^2) between selected SNPs in *MDM4* gene

SNPs	rs3789051	rs4245739	rs16853949	rs10900598
rs3789051	--	0.713	0.632	0.552
rs4245739	0.114	--	0.794	0.695
rs16853949	0.033	0.252	--	0.482
rs10900598	0.012	0.041	0.003	--

D’ values are given above the diagonal; r^2 values are given below the diagonal.

Supplementary Table S6: Association between *MDM4* haplotypes and OS in Discovery set

Haplotype	Frequency	N (%)	mOS (95% CI) (m) [a]	P_{L-R}	aHR (95% CI) [b]	P [b]
**G-A-A-A**	0.476					
Copy number 0		138 (21.5)	21.05 (17.78-24.26)	0.089	Ref.	
Copy number 1-2		504 (78.5)	18.27 (14.82-21.07)		1.22 (0.84-1.69)	0.172
**G-A-C-A**	0.295					
Copy number 0		216 (33.6)	20.40 (18.93-22.87)	0.352	Ref.	
Copy number 1-2		426 (66.4)	18.90 (16.14-20.66)		1.09 (0.76-1.33)	0.441
**A-A-C-C**	0.108					
Copy number 0		563 (87.7)	19.53 (17.28-22.08)	0.573	Ref.	
Copy number 1-2		79 (12.3)	20.17 (17.86-23.62)		0.90 (0.75-1.14)	0.628

OS overall survival, m months, Ref. reference, P_{L-R} Log-Rank P, HR hazard ratio, CI confidence interval;

[a] survival derived from Kaplan–Meier analysis;

[b] HRs, 95% CIs and their corresponding *p*-values were calculated using multivariate Cox proportional hazard models, adjusted for all clinical factors.
