# Supplementary material for: Variations within 3′-UTR of MDM4 gene contribute to clinical outcomes of advanced non-small cell lung cancer patients following platinum-based chemotherapy
Source: Oncotarget. 2016 Jul 22;8(10):16313–24. doi: 10.18632/oncotarget.10771 (PMC5369965; doi:10.18632/oncotarget.10771)
Supplement: Supplementary file 2 [file oncotarget-08-16313-s002.docx]

**Table S1:** **Distribution of *MDM4* genotypes according to clinical factors in Discovery set**

| **Variables** | **rs3789051, N** | | | |  | **rs4245739, N** | | | | |  | **rs16853949, N** | | | |  | **rs10900598, N** | | | |
| --- | --- | --- | --- | --- | --- | --- | --- | --- | --- | --- | --- | --- | --- | --- | --- | --- | --- | --- | --- | --- |
|  | GG | AG | AA | *P ^a^* |  | AA | AC | | CC | *P ^a^* |  | CC | AC | AA | *P ^a^* |  | AA | AC | CC | *P ^a^* |
| Age (years) |  |  |  |  |  |  |  |  | |  |  |  |  |  |  |  |  |  |  |  |
| < 58 (n=333) | 169 | 137 | 27 | 0.304 |  | 297 | 32 | 4 | | 0.306 |  | 252 | 56 | 23 | 0.968 |  | 293 | 27 | 12 | 0.884 |
| ≥ 58 (n=309) | 171 | 121 | 17 |  |  | 271 | 29 | 9 | |  |  | 233 | 52 | 23 |  |  | 269 | 28 | 10 |  |
| Gender |  |  |  |  |  |  |  |  | |  |  |  |  |  |  |  |  |  |  |  |
| Male (n=456) | 244 | 178 | 34 | 0.486 |  | 408 | 40 | 8 | | 0.443 |  | 341 | 81 | 34 | 0.574 |  | 395 | 39 | 19 | 0.265 |
| Female (n=186) | 96 | 80 | 10 |  |  | 160 | 21 | 5 | |  |  | 144 | 27 | 12 |  |  | 167 | 16 | 3 |  |
| Smoking history |  |  |  |  |  |  |  |  | |  |  |  |  |  |  |  |  |  |  |  |
| Non-smokers (n=260) | 133 | 109 | 18 | 0.741 |  | 228 | 26 | 6 | | 0.853 |  | 203 | 37 | 17 | 0.310 |  | 234 | 21 | 4 | 0.083 |
| Smokers (n=382) | 207 | 149 | 26 |  |  | 340 | 35 | 7 | |  |  | 282 | 71 | 29 |  |  | 328 | 34 | 18 |  |
| ECOG PS |  |  |  |  |  |  |  |  | |  |  |  |  |  |  |  |  |  |  |  |
| 0-1 (n=593) | 315 | 236 | 42 | 0.630 |  | 523 | 58 | 12 | | 0.703 |  | 450 | 100 | 41 | 0.667 |  | 517 | 52 | 21 | 0.679 |
| 2 (n=49) | 25 | 22 | 2 |  |  | 45 | 3 | 1 | |  |  | 35 | 8 | 5 |  |  | 45 | 3 | 1 |  |
| Chemotherapy |  |  |  |  |  |  |  |  | |  |  |  |  |  |  |  |  |  |  |  |
| NP/NC (n=236) | 125 | 92 | 19 | 0.140 |  | 214 | 15 | 7 | | 0.103 |  | 188 | 28 | 19 | 0.124 |  | 215 | 16 | 5 | 0.281 |
| GP/GC (n=174) | 86 | 82 | 6 |  |  | 148 | 21 | 5 | |  |  | 128 | 30 | 15 |  |  | 150 | 19 | 5 |  |
| TP/TC (n=192) | 106 | 72 | 14 |  |  | 170 | 22 | 0 | |  |  | 139 | 42 | 11 |  |  | 162 | 18 | 9 |  |
| DP/DC (n=40) | 23 | 12 | 5 |  |  | 36 | 3 | 1 | |  |  | 30 | 8 | 1 |  |  | 35 | 2 | 3 |  |
| TNM stage |  |  |  |  |  |  |  |  | |  |  |  |  |  |  |  |  |  |  |  |
| III (n=262) | 133 | 110 | 19 | 0.650 |  | 226 | 27 | 9 | | 0.087 |  | 206 | 42 | 13 | 0.156 |  | 223 | 22 | 14 | 0.080 |
| IV (n=380) | 207 | 148 | 25 |  |  | 342 | 34 | 4 | |  |  | 279 | 66 | 33 |  |  | 339 | 33 | 8 |  |
| Histological type |  |  |  |  |  |  |  |  | |  |  |  |  |  |  |  |  |  |  |  |
| Adeno (n=398) | 211 | 154 | 33 | 0.287 |  | 348 | 43 | 7 | | 0.112 |  | 297 | 64 | 34 | 0.473 |  | 350 | 36 | 11 | 0.144 |
| SQC (n=147) | 82 | 59 | 6 |  |  | 138 | 6 | 3 | |  |  | 115 | 25 | 7 |  |  | 128 | 8 | 9 |  |
| Others (n=97) | 47 | 45 | 5 |  |  | 82 | 12 | 3 | |  |  | 73 | 19 | 5 |  |  | 84 | 11 | 2 |  |

Adeno adenocarcinoma, SQC squamous cell carcinoma, N number, ^a^ *p*-values derived from *χ*^2^ test.
