# Supplementary material for: Variations within 3′-UTR of MDM4 gene contribute to clinical outcomes of advanced non-small cell lung cancer patients following platinum-based chemotherapy
Source: Oncotarget. 2016 Jul 22;8(10):16313–24. doi: 10.18632/oncotarget.10771 (PMC5369965; doi:10.18632/oncotarget.10771)
Supplement: Supplementary file 3 [file oncotarget-08-16313-s003.docx]

**Table S2:** **Clinical characteristics associated with OS and PFS of patients in each set and in pooled populations**

| **Variables** | **Discovery set** | | | | | |  | **Replication set** | | | | | |  | **Pooled** | | | | | | | | | |  |
| --- | --- | --- | --- | --- | --- | --- | --- | --- | --- | --- | --- | --- | --- | --- | --- | --- | --- | --- | --- | --- | --- | --- | --- | --- | --- |
|  | OS | | | PFS | | |  | OS | | | PFS | | |  | OS | | | | | | PFS | | | |  |
|  | N | aHR (95% CI) | *P* | N | aHR (95% CI) | *P* |  | N | aHR (95% CI) | *P* | N | aHR (95% CI) | *P* |  | N | | aHR (95% CI) | | *P* | | N | aHR (95% CI) | *P* | |  |
| Age (years) |  |  |  |  |  |  |  |  |  |  |  |  |  |  |  | |  | |  | |  |  |  | |  |
| < 58 | 333 | Ref. |  | 306 | Ref. |  |  | 171 | Ref. |  | 170 | Ref. |  |  | 504 | | Ref. | |  | | 476 | Ref. |  | |  |
| ≥ 58 | 309 | 1.17 (0.98-1.41) | 0.095 | 283 | 0.98 (0.79-1.21) | 0.852 |  | 159 | 1.30 (0.99-1.70) | 0.056 | 156 | 0.99 (0.76-1.29) | 0.961 |  | 468 | | 1.20 (1.03-1.40) | | 0.017 | | 439 | 0.97 (0.82-1.14) | 0.700 | |  |
| Gender |  |  |  |  |  |  |  |  |  |  |  |  |  |  |  | |  | |  | |  |  |  | |  |
| Male | 456 | Ref. |  | 413 | Ref. |  |  | 234 | Ref. |  | 231 | Ref. |  |  | | 690 | | Ref. | |  | 644 | Ref. | |  | |
| Female | 186 | 0.88 (0.66-1.18) | 0.375 | 176 | 1.28 (0.90-1.81) | 0.174 |  | 96 | 0.82 (0.56-1.21) | 0.322 | 95 | 0.79 (0.53-1.16) | 0.224 |  | 282 | | 0.87 (0.69-1.10) | | 0.233 | | 271 | 1.06 (0.82-1.38) | 0.245 | |  |
| Smoking history |  |  |  |  |  |  |  |  |  |  |  |  |  |  |  | |  | |  | |  |  |  | |  |
| Non-smokers | 260 | Ref. |  | 240 | Ref. |  |  | 144 | Ref. |  | 143 | Ref. |  |  | 404 | | Ref. | |  | | 383 | Ref. |  | |  |
| Smokers | 382 | 1.03 (0.79-1.35) | 0.858 | 349 | 1.10 (0.79-1.52) | 0.572 |  | 186 | 1.06 (0.75-1.50) | 0.740 | 183 | 0.82 (0.57-1.16) | 0.262 |  | 568 | | 1.05 (0.85-1.29) | | 0.678 | | 532 | 0.99 (0.79-1.26) | 0.857 | |  |
| ECOG PS |  |  |  |  |  |  |  |  |  |  |  |  |  |  |  | |  | |  | |  |  |  | |  |
| 0-1 | 593 | Ref. |  | 546 | Ref. |  |  | 297 | Ref. |  | 293 | Ref. |  |  | 890 | | Ref. | |  | | 839 | Ref. |  | |  |
| 2 | 49 | 1.24 (0.89-1.73) | 0.206 | 43 | 1.70 (1.16-2.49) | 0.006 |  | 33 | 1.21 (0.77-1.89) | 0.409 | 33 | 1.39 (0.97-1.85) | 0.032 |  | 82 | | 1.22 (0.94-1.59) | | 0.134 | | 76 | 1.54 (1.16-2.04) | 0.003 | |  |
| Chemotherapy |  |  |  |  |  |  |  |  |  |  |  |  |  |  |  | |  | |  | |  |  |  | |  |
| NP/NC | 236 | Ref. |  | 231 | Ref. |  |  | 115 | Ref. |  | 115 | Ref. |  |  | 351 | | Ref. | |  | | 346 | Ref. |  | |  |
| GP/GC | 174 | 1.06 (0.72-1.55) | 0.778 | 154 | 1.36 (0.83-1.78) | 0.129 |  | 95 | 1.13 (0.73-1.78) | 0.570 | 95 | 0.94 (0.68-1.30) | 0.706 |  | 269 | | 0.95 (0.79-1.14) | | 0.549 | | 249 | 1.19 (0.96-1.46) | 0.106 | |  |
| TP/TC | 192 | 1.19 (0.96-1.49) | 0.116 | 175 | 1.33 (0.95-1.71) | 0.232 |  | 95 | 1.11 (0.80-1.56) | 0.534 | 93 | 1.10 (0.80-1.52) | 0.561 |  | 287 | | 1.01 (0.85-1.22) | | 0.881 | | 268 | 1.23 (1.00-1.50) | 0.347 | |  |
| DP/DC | 40 | 0.93 (0.74-1.17) | 0.381 | 29 | 1.03 (0.61-1.75) | 0.806 |  | 25 | 1.22 (0.87-1.70) | 0.243 | 23 | 1.10 (0.62-1.94) | 0.745 |  | 65 | | 0.81 (0.59-1.12) | | 0.199 | | 52 | 1.08 (0.74-1.57) | 0.704 | |  |
| TNM stage |  |  |  |  |  |  |  |  |  |  |  |  |  |  |  | |  | |  | |  |  |  | |  |
| III | 262 | Ref. |  | 234 | Ref. |  |  | 119 | Ref. |  | 118 | Ref. |  |  | 381 | | Ref. | |  | | 352 | Ref. |  | |  |
| IV | 380 | 1.28 (1.06-1.54) | 0.012 | 355 | 1.19 (0.95-1.49) | 0.128 |  | 211 | 1.40 (1.07-1.84) | 0.015 | 208 | 1.05 (0.80-1.38) | 0.726 |  | 591 | | 1.30 (1.12-1.52) | | 0.001 | | 563 | 1.14 (0.96-1.35) | 0.138 | |  |
| Histological type |  |  |  |  |  |  |  |  |  |  |  |  |  |  |  | |  | |  | |  |  |  | |  |
| Adeno | 398 | Ref. |  | 369 | Ref. |  |  | 220 | Ref. |  | 217 | Ref. |  |  | 618 | | Ref. | |  | | 586 | Ref. |  | |  |
| SQC | 147 | 1.26 (0.99-1.60) | 0.053 | 130 | 1.16 (0.84-1.42) | 0.587 |  | 66 | 1.27 (0.89-1.68) | 0.192 | 66 | 0.75 (0.52-1.08) | 0.120 |  | 213 | | 1.16 (0.96-1.42) | | 0.126 | | 196 | 0.97 (0.76-1.25) | 0.631 | |  |
| Others | 97 | 1.35 (1.03-1.76) | 0.027 | 90 | 1.22 (0.97-1.66) | 0.052 |  | 44 | 1.60 (1.08-2.35) | 0.018 | 43 | 1.03 (0.69-1.52) | 0.891 |  | 141 | | 1.37 (1.10-1.70) | | 0.005 | | 133 | 1.08 (0.87-1.35) | 0.468 | |  |

OS overall survival, PFS progression-free survival, Adeno adenocarcinoma, SQC squamous cell carcinoma, HR hazard ratio, CI confidence interval, *p*-values were calculated using multivariate Cox proportional hazard models adjusted for all clinical factors.
