# Supplementary material for: Variations within 3′-UTR of MDM4 gene contribute to clinical outcomes of advanced non-small cell lung cancer patients following platinum-based chemotherapy
Source: Oncotarget. 2016 Jul 22;8(10):16313–24. doi: 10.18632/oncotarget.10771 (PMC5369965; doi:10.18632/oncotarget.10771)
Supplement: Supplementary file 4 [file oncotarget-08-16313-s004.docx]

**Table S3:** **OS and PFS in relation to *MDM4* genotype distributions in Discovery set**

| **SNPs** | **OS** | | | | |  | **PFS** | | | | |
| --- | --- | --- | --- | --- | --- | --- | --- | --- | --- | --- | --- |
|  | N (%) | mOS (95% CI) (m) ^a^ | *P*_L-R_ | aHR (95% CI) ^b^ | *P* ^b^ |  | N (%) | mPFS (95% CI) (m) ^a^ | *P*_L-R_ | aHR (95% CI) ^b^ | *P* ^b^ |
| **rs3789051** |  |  |  |  |  |  |  |  |  |  |  |
| GG | 340 (53.0) | 18.23 (16.02-20.45) | 0.844 | Ref. |  |  | 313 (53.1) | 10.50 (8.28-12.72) | 0.204 | Ref. |  |
| AG | 258 (40.2) | 19.83 (17.48-22.19) |  | 0.92 (0.78-1.21) | 0.944 |  | 236 (40.1) | 9.10 (7.15-11.05) |  | 1.14 (0.92-1.42) | 0.235 |
| AA | 44 (6.9) | 21.23 (18.16-24.31) |  | 0.84 (0.65-1.25) | 0.742 |  | 40 (6.8) | 11.93 (2.93-20.93) |  | 0.89 (0.56-1.42) | 0.627 |
| **rs4245739** |  |  |  |  |  |  |  |  |  |  |  |
| AA | 568 (88.5) | 18.67 (16.83-20.50) | 0.015 | Ref. |  |  | 521 (88.5) | 9.67 (8.20-11.15) | 0.302 | Ref. |  |
| AC | 61 (9.5) | 21.27 (15.07-27.47) |  | 0.71 (0.52-0.98) | 0.037 |  | 56 (9.5) | 13.00 (3.71-22.29) |  | 0.74 (0.51-1.08) | 0.115 |
| CC | 13 (2.0) | 31.27 (26.49-36.05) |  | 0.50 (0.25-1.02) | 0.055 |  | 12 (2.0) | 10.40 (3.66-17.14) |  | 1.10 (0.54-2.25) | 0.795 |
| **rs16853949** |  |  |  |  |  |  |  |  |  |  |  |
| CC | 485 (75.5) | 18.83 (16.85-20.82) | 0.880 | Ref. |  |  | 439 (74.5) | 9.83 (8.09-11.57) | 0.951 | Ref. |  |
| AC | 108 (16.8) | 19.90 (16.19-23.61) |  | 0.95 (0.74-1.21) | 0.648 |  | 102 (17.3) | 9.80 (6.34-13.26) |  | 0.95 (0.71-1.26) | 0.713 |
| AA | 46 (7.2) | 19.53 (9.11-29.95) |  | 0.90 (0.62-1.29) | 0.558 |  | 45 (7.6) | 11.00 (3.99-18.01) |  | 0.92 (0.61-1.37) | 0.662 |
| **rs10900598** |  |  |  |  |  |  |  |  |  |  |  |
| AA | 476 (74.1) | 17.80 (15.44-20.16) | 0.017 | Ref. |  |  | 431 (73.2) | 9.63 (8.07-11.19) | 0.429 | Ref. |  |
| AC | 115 (17.9) | 20.00 (17.45-22.55) |  | 0.86 (0.68-1.10) | 0.227 |  | 109 (18.5) | 10.07 (6.03-14.11) |  | 0.93 (0.70-1.23) | 0.619 |
| CC | 49 (7.6) | 25.77 (15.92-35.61) |  | 0.65 (0.46-0.93) | 0.017 |  | 47 (8.0) | 16.47 (2.24-30.70) |  | 0.90 (0.61-1.33) | 0.602 |

OS overall survival, PFS progression-free survival, m months, Ref. reference, HR hazard ratio, CI confidence interval, *P*_L-R_ Log-Rank *P*;

^a^ survival derived from Kaplan–Meier analysis;

^b^ HRs, 95% CIs and their corresponding *p*-values were calculated using multivariate Cox proportional hazard models, adjusted for all clinical factors.
