# Supplementary material for: Variations within 3′-UTR of MDM4 gene contribute to clinical outcomes of advanced non-small cell lung cancer patients following platinum-based chemotherapy
Source: Oncotarget. 2016 Jul 22;8(10):16313–24. doi: 10.18632/oncotarget.10771 (PMC5369965; doi:10.18632/oncotarget.10771)
Supplement: Supplementary file 5 [file oncotarget-08-16313-s005.docx]

**Table S4: Association between *MDM4* SNPs and OS in Discovery set stratified by tumor histology**

| **SNPs** | **Adenocarcinoma** | | | | |  | **Squamous cell carcinoma** | | | | |  | **Others** | | | | |
| --- | --- | --- | --- | --- | --- | --- | --- | --- | --- | --- | --- | --- | --- | --- | --- | --- | --- |
|  | N | mOS (95%CI) (m) ^a^ | *P*_L-R_ | aHR (95% CI) ^b^ | *P* ^b^ |  | N | mOS (95%CI) (m) ^a^ | *P*_L-R_ | aHR (95% CI) ^b^ | *P* ^b^ |  | N | mOS (95%CI) (m) ^a^ | *P*_L-R_ | aHR (95% CI) ^b^ | *P* ^b^ |
| **rs10900598** |  |  |  |  |  |  |  |  |  |  |  |  |  |  |  |  |  |
| AA | 297 | 19.10 (16.74-21.46) | 0.039 | Ref. |  |  | 110 | 13.70 (8.30-19.20) | 0.476 | Ref. |  |  | 69 | 14.03 (10.41-17.66) | 0.723 | Ref. |  |
| AC | 67 | 22.40 (17.45-27.35) |  | 0.77 (0.56-1.05) | 0.103 |  | 27 | 15.00 (6.74-23.27) |  | 0.97 (0.58-1.63) | 0.911 |  | 21 | 17.63 (9.12-26.14) |  | 0.94 (0.54-1.88) | 0.989 |
| CC | 33 | 31.20 (22.40-40.00) |  | 0.64 (0.41-1.00) | 0.048 |  | 9 | 22.17 (13.13-31.20) |  | 0.74 (0.31-1.74) | 0.487 |  | 7 | 25.37 (13.73-37.00) |  | 0.52 (0.21-1.30) | 0.165 |
| dominant |  |  |  |  |  |  |  |  |  |  |  |  |  |  |  |  |  |
| CC+AC | 100 | 24.53 (17.10-31.96) | 0.013 | 0.73 (0.55-0.95) | 0.020 |  | 36 | 19.53 (13.40-25.67) | 0.326 | 0.91 (0.57-1.45) | 0.681 |  | 28 | 19.93 (11.42-28.44) | 0.490 | 0.80 (0.47-1.37) | 0.419 |
| AA | 297 | 19.10 (16.74-21.46) |  | Ref. |  |  | 110 | 13.70 (8.30-19.20) |  | Ref. |  |  | 69 | 14.03 (10.41-17.66) |  | Ref. |  |
| recessive |  |  |  |  |  |  |  |  |  |  |  |  |  |  |  |  |  |
| CC | 33 | 31.20 (22.40-40.00) | 0.076 | 0.68 (0.44-1.05) | 0.082 |  | 9 | 22.17 (13.13-31.20) | 0.268 | 0.74 (0.32-1.74) | 0.492 |  | 7 | 25.37 (13.73-37.00) | 0.481 | 0.52 (0.21-1.30) | 0.162 |
| AA+AC | 364 | 19.83 (17.86-21.81) |  | Ref. |  |  | 137 | 14.27 (9.61-18.93) |  | Ref. |  |  | 90 | 15.07 (11.26-18.88) |  | Ref. |  |
| additive |  | NA | NA | 0.79 (0.65-0.96) | 0.017 |  |  | NA | NA | 0.90 (0.63-1.28) | 0.549 |  |  | NA | NA | 0.80 (0.55-1.16) | 0.238 |
| **rs4245739** |  |  |  |  |  |  |  |  |  |  |  |  |  |  |  |  |  |
| AA | 348 | 19.90 (17.70-22.10) | 0.042 | Ref. |  |  | 138 | 15.00 (10.41-19.59) | 0.154 | Ref. |  |  | 82 | 15.07 (12.05-18.08) | 0.469 | Ref. |  |
| AC | 43 | 31.27 (11.53-51.01) |  | 0.67 (0.46-0.99) | 0.047 |  | 6 | 12.53 (6.66-21.46) |  | 1.71 (0.72-4.06) | 0.225 |  | 12 | 22.30 (12.17-36.14) |  | 0.59 (0.28-1.28) | 0.181 |
| CC | 7 | 32.70 (13.54-51.86) |  | 0.51 (0.19-1.37) | 0.180 |  | 3 | 29.87 (23.31-36.43) |  | 0.39 (0.10-1.62) | 0.195 |  | 3 | 33.47 () |  | 0.37 (0.08-1.68) | 0.199 |
| dominant |  |  |  |  |  |  |  |  |  |  |  |  |  |  |  |  |  |
| CC+AC | 50 | 32.70 (17.90-47.50) | 0.013 | 0.65 (0.45-0.93) | 0.020 |  | 9 | 20.67 (18.04-23.30) | 0.886 | 0.93 (0.45-1.93) | 0.844 |  | 15 | 22.30 (7.07-37.53) | 0.236 | 0.54 (0.26-1.12) | 0.097 |
| AA | 348 | 19.90 (17.70-22.10) |  | Ref. |  |  | 138 | 15.00 (10.41-19.59) |  | Ref. |  |  | 82 | 15.07 (12.05-18.08) |  | Ref. |  |
| additive |  | NA | NA | 0.69 (0.50-0.94) | 0.019 |  |  | NA | NA | 0.83 (0.50-1.36) | 0.456 |  |  | NA | NA | 0.60 (0.34-1.07) | 0.085 |

OS overall survival, m months, Ref. reference, NA not available, HR hazard ratio, CI confidence interval, *P*_L-R_ Log-Rank *P*;

^a^ survival derived from Kaplan–Meier analysis;

^b^ HRs, 95% CIs and their corresponding *p*-values were calculated using multivariate Cox proportional hazard models, adjusted for all clinical factors.
